# Chronic Pharyngitis

**Published:** 1867-01

**Authors:** J. R. Black

**Affiliations:** Newark, O.


*Chronic Pharyngitis.* By J. R. Black, M. D., Newark, O.

Too true is it, that the majority of physicians give anxious thought and care only to diseases of a grave or very painful character. Even in our journals, the minor ills (of which chronic pharyngitis is a fair example) are almost wholly ignored. Like many cutaneous affections, it is neither dangerous nor painful, but yet how annoying its existence often is to the physician, as well as the victim. The honorable and the honest among our patrons, mingle esteem and gratitude, with their dollars, to him who rids them of such troublesome and disturbing pests.

It is perfectly true that the duties of the physician are so numerous, so diverse, and so complex, that long and careful study, cultivated and critical observation, logical mind, and a very retentive memory, are essential requisites to great excellence and skill in every department of this, our wide field. It is the very few who possess, to a marked degree, such unique endowments, and there is therefore much to apologize for our imperfections. The only censure that can rightfully attach to our shortcomings is when carelessness, neglect, or conceit hinders, or altogether prevents our improvement. It is not of will, or of province, to act the censor; but this may be said of those too indolent to read, and learn that they deserve pity: and those whose greatness and self-rated importance have swelled up within them so as to preclude any ideal endosmosis, that they can not command love, respect, or veneration. Thankful on my part for what the periodical press, with its thousands of noble contributors—laboring for the common and God-like weal—have done for me, I shall endeavor to give my mite, in small requital, in this, as in other efforts.

To describe the lingering inflammation which is of commonly seen upon one or both tonsils, and along the pillars of the soft palate, would be entirely superfluous. Its extension also down the pharynx, or up into the posterior nares, are familiar facts. Its various stages and degrees are also to be daily seen, running from a simple smooth redness and swelling, to follicular, and honeycomb ulceration. The amygdalæ, especially in scrofulous subjects, are more or less permanently enlarged—in many instances almost blocking up the isthmus of the fauces. The common effects are stiffness and soreness of the parts, often slight pain in deglutition, and a discharge, by hawking, of more or less glary mucus, frequently mingled with pus cells. The constant irritation and expectoration are very commonly aggravated by every slight exposure, and at such times there is usually a manifest disposition of the disease to extend downwards upon the glottis into the larynx and trachea. The symptoms in this event are a slight hoarseness, an ever recurring effort at swallowing, and an effort to clear the throat, with cough, and a rough soreness along and behind the sternum.

The young practitioner who relies upon the text-books as guides in the treatment of chronic pharyngitis, will commonly find his mind very much afloat as to what it is best to do. If he has studied the teachings of Dr. Horace Green, the topical treatment by nitras argenti will probably be quickly adopted. If those of Prof. Flint, he will endeavor to follow his precept of making "the object of treatment, in short, to restore the general health," whatever that may mean. One authority thus relies mainly upon the topical, the other upon the general treatment. There is doubtless truth in both systems. When the general health is toned high enough, the continuance of a minor ailment like this is rendered impossible. But such toning is very seldom possible, or practicable. The very existence shows a state of the system, or a local predisposition, fundamentally faulty. This may be inherent, or developed. If the former, then we can not hope to remove it; if the latter, then there is hope in hygienic rules. But the disease is often found in persons whose general health appears otherwise unexceptionable; and in such cases what is the course to be pursued? In a majority of cases, the most persevering use of the nitrate of silver does not satisfactorily control, much less cure the disease. At least such has been the experience of the writer, and to those whose observation on this point has been identical, the following treatment is commended to their attention:

In those examples in which there is no obvious general indication to fill, one or the other of the applications to be mentioned have rarely failed to give prompt relief, and when persevered in to effect a radical cure. ℞ Tinct. Iodine, Inod. Glycerine, aa ℥ ss., Bals. Fir. ℥ j ss. Apply to the irritated or ulcerated parts, once daily, with a camel's hair brush. This preparation diffuses itself rapidly over the fauces, soothing the irritation, and clearing the throat by free expectoration. When the inflammation has extended into the nasal cavity, the most convenient and practicable mode of reaching it with the medicine is by insufflation. Pour half a teaspoonful into the palm of the hand, or on a bit of sized paper, apply closely to the nostril, close the opposite one with the finger, and give a forcible inspiration. In case the disease had extended into the larynx, and become chronic, the tincture of iodine mixed with spirits of ether, comp. and used by inhaling the vapor, gives very gratifying results. A very good inhaler can be extemporized from a quinine bottle. Fit two good quills into a tight cork, one end of one extending an inch or two into the liquid. To the superior end of the one not dipping the liquid, attach a gum elastic bougie, with a mouth-piece. This may be used once or twice daily.

From some idiosyncrasy, or other inexplicable reason, the above remedy does not always have its usual curative virtues, in which instances the following elegant preparation will be found strikingly beneficial. The active ingredient is the same used in Luly's patent nostrum. ℞ Hyd. bichloride, grs. viii.; Ammon. murias, grs. xx.; Inod. glycerine; Aqua rose, aa. ℥ ss. M. Apply as above, though greater caution is required against swallowing any of the mixture.

The immediate effect of either remedy is an amelioration of the more prominent symptoms, and to insure a radical cure, the main point is to use them perseveringly. When in young subjects, the marked scrofulous diathesis is associated with an all but permanent enlargement of the tonsils, great benefit will be derived from the following internal remedy: ℞ Fl. ex. Lappa Maj.; Fl. ex. Revmex obt. aa. ℥ j.; Tr. G. Guiac, ℥ ss; Tr. Columbo, ℥ j. M. Teaspoonful thrice daily. In this way the necessity of their excision may be frequently obviated.—*Cin. Lancet.*

